# Deep histopathology genotype–phenotype analysis of focal cortical dysplasia type II differentiates between the GATOR1-altered autophagocytic subtype IIa and *MTOR*-altered migration deficient subtype IIb

**DOI:** 10.1186/s40478-023-01675-x

**Published:** 2023-11-09

**Authors:** Jonas Honke, Lucas Hoffmann, Roland Coras, Katja Kobow, Costin Leu, Tom Pieper, Till Hartlieb, Christian G. Bien, Friedrich Woermann, Thomas Cloppenborg, Thilo Kalbhenn, Ahmed Gaballa, Hajo Hamer, Sebastian Brandner, Karl Rössler, Arnd Dörfler, Stefan Rampp, Johannes R. Lemke, Sara Baldassari, Stéphanie Baulac, Dennis Lal, Peter Nürnberg, Ingmar Blümcke

**Affiliations:** 1 Department of Neuropathology, Universitätsklinikum Erlangen, FAU Erlangen-Nürnberg, Erlangen, Germany; 2 Partner of the European Reference Network (ERN) EpiCARE, Barcelona, Spain; 3 Genomic Medicine Institute, Lerner Research Institute, Cleveland Clinic, Cleveland, OH 44195 USA; 4 Charles Shor Epilepsy Center, Neurological Institute, Cleveland Clinic, Cleveland, USA; 5 Stanley Center for Psychiatric Research, Broad Institute of Harvard and M.I.T, Cambridge, MA 02142 USA; 6 Cologne Center for Genomics (CCG), Medical Faculty of the University of Cologne, University Hospital of Cologne, 50931 Cologne, Germany; 7 Center for Pediatric Neurology, Neurorehabilitation, and Epileptology, Schoen-Clinic, Vogtareuth, Germany; 8 Research Institute for Rehabilitation, Transition, and Palliation, Paracelsus Medical University, Salzburg, Austria; 9 Department of Epileptology (Krankenhaus Mara), Medical School, Bielefeld University, Bielefeld, Germany; 10 Department of Neurosurgery (Evangelisches Klinikum Bethel), Medical School, Bielefeld University, Bielefeld, Germany; 11 Epilepsy Center, Universitätsklinikum Erlangen, FAU Erlangen-Nürnberg, Erlangen, Germany; 12 Department of Neurosurgery, Universitätsklinikum Erlangen, FAU Erlangen-Nürnberg, Erlangen, Germany; 13 Department of Neurosurgery, Medical University of Vienna, Vienna General Hospital, Vienna, Austria; 14 Department of Neuroradiology, Universitätsklinikum Erlangen, FAU Erlangen-Nürnberg, Erlangen, Germany; 15 Inserm, CNRS, APHP, Institut du Cerveau - Paris Brain Institute - ICM, Hôpital de La Pitié Salpêtrière, Sorbonne Université, Paris, France; 16 Institute of Human Genetics, University of Leipzig Medical Center, Leipzig, Germany; 17 Center for Rare Diseases, University of Leipzig Medical Center, Leipzig, Germany; 18 Department of Clinical and Experimental Epilepsy, Institute of Neurology, University College London, London, UK; 19 Department of Neurology, McGovern Medical School, UTHealth Houston, University of Texas, Houston, USA

## Abstract

Focal cortical dysplasia type II (FCDII) is the most common cause of drug-resistant focal epilepsy in children. Herein, we performed a deep histopathology-based genotype–phenotype analysis to further elucidate the clinico-pathological and genetic presentation of FCDIIa compared to FCDIIb. Seventeen individuals with histopathologically confirmed diagnosis of FCD ILAE Type II and a pathogenic variant detected in brain derived DNA whole-exome sequencing or mTOR gene panel sequencing were included in this study. Clinical data were directly available from each contributing centre. Histopathological analyses were performed from formalin-fixed, paraffin-embedded tissue samples using haematoxylin–eosin and immunohistochemistry for NF-SMI32, NeuN, pS6, p62, and vimentin. Ten individuals carried loss-of-function variants in the GATOR1 complex encoding genes *DEPDC5* (n = 7) and *NPRL3* (n = 3), or gain-of-function variants in *MTOR* (n = 7). Whereas individuals with GATOR1 variants only presented with FCDIIa, i.e., lack of balloon cells, individuals with *MTOR* variants presented with both histopathology subtypes, FCDIIa and FCDIIb. Interestingly, 50% of GATOR1-positive cases showed a unique and predominantly vacuolizing phenotype with p62 immunofluorescent aggregates in autophagosomes. All cases with GATOR1 alterations had neurosurgery in the frontal lobe and the majority was confined to the cortical ribbon not affecting the white matter. This pattern was reflected by subtle or negative MRI findings in seven individuals with GATOR1 variants. Nonetheless, all individuals were seizure-free after surgery except four individuals carrying a *DEPDC5* variant. We describe a yet underrecognized genotype–phenotype correlation of GATOR1 variants with FCDIIa in the frontal lobe. These lesions were histopathologically characterized by abnormally vacuolizing cells suggestive of an autophagy-altered phenotype. In contrast, individuals with FCDIIb and brain somatic *MTOR* variants showed larger lesions on MRI including the white matter, suggesting compromised neural cell migration.

**Supplementary Information:**

The online version contains supplementary material available at 10.1186/s40478-023-01675-x.

## Introduction

Histopathological assessment of epilepsy surgery human brain specimens revealed focal cortical dysplasia ILAE type II (FCDII) as the single most common cause of drug-resistant focal epilepsy in children and the third most common in adults [12]. Almost all individuals with FCDII present with epileptic seizures at an early age and continuous rhythmic spiking in electroencephalography [14, 48, 63]. The ILAE classification scheme for FCD further separates subtype IIa with dysmorphic neurons from IIb with dysmorphic neurons and balloon cells [13, 44]. The co-registration of intracerebral electroencephalography with histopathology suggested dysmorphic neurons as the cellular source for interictal and ictal neurophysiological events in FCDII [53]. This correlation was not reported for balloon cells, which is another important signature cell population in FCDIIb [15, 53]. Dysmorphic neurons are microscopically defined by abnormal orientation, enlarged cell bodies, cytoplasmic accumulation of neurofilament proteins and pS6 immunoreactivity indicating constitutive activation of the mTOR signalling pathway [26, 27]. Indeed, most published cases with histopathologically confirmed FCDII can be genetically defined by brain somatic mosaicism of mTOR signalling pathway genes [9, 24]. Pathogenic variants in *AKT3*, *DEPDC5, MTOR, NPRL2*, *NPRL3*, *PIK3CA*, *PTEN*, *RHEB, TSC1,* and *TSC2* have been reported previously [2, 17, 33, 35, 40, 47, 51]. The diagnostic yield of such mTOR-related variants in all cortical dysplasias is in the range of 20–80% of individuals, mainly depending on the histopathology subtype (hemimegalencephaly > FCDII), diagnostic genetic testing methods (digital droplet PCR > panel sequencing) and access to histopathologically characterized tissue before extracting DNA [24]. Notably, laser microdissection experiments could allocate these pathogenic variants directly to the affected population of dysmorphic neurons and balloon cells [2, 36].

The most commonly recognized brain somatic variants in FCDII are affecting *MTOR* [34, 37, 47], especially in FCDIIb [21, 45, 61]. Gain-of-function *MTOR* variants activate the mTOR complex 1, a central module of the mTOR signalling pathway, regulating cell growth [28, 32, 39], survival [28, 50] and migration [1]. Experimental in-utero electroporation of gain-of-function *MTOR* variants into the developing mouse cortex cause cytomegalic, pS6 immunoreactive neurons, cortical dyslamination and intractable epilepsy [37]. In contrast, most *DEPDC5* variants are loss-of-function germline variants [4, 20, 22]. Together with *NPRL2* and *NPRL3*, *DEPDC5* is part of the GATOR1 complex which senses the amino acid content of the cell as a negative regulator of mTORc1 [3]. As confirmed in independent individual series, an additional brain somatic second hit in *DEPDC5* is likely necessary to constitutively activate mTORC1 in the affected tissue [2, 4, 36, 40, 54, 56].

Despite advances in our understanding of FCDII, diagnostic methods and disease classification schemes, recognition of FCDII subtypes remains challenging in clinical practice [13, 44, 49, 57]. Magnetic resonance imaging (MRI) techniques became most helpful in identifying these cortical malformations [5], which range in size from hemispheric dysplasias, i.e. hemimegalencephaly, to subtle bottom-of-sulcus FCDII [21, 63]. Postsurgical outcomes often correlate with the visibility of the lesion by MRI, as it compromises and delays the decision-making for surgical treatment and completeness of the surgical resection field when MRI is negative [59, 62]. Herein, we performed a deep histopathology-based genotype–phenotype analysis to study the value of an integrated molecular, histopathology and clinical diagnosis of FCDIIa and IIb subtypes as recently suggested by ILAE’s FCD classification update from 2022 [44].

## Material and methods

### Individuals included in this study

We included 17 individuals (8 female, 9 male, Table 1) with drug-resistant focal epilepsy, who underwent epilepsy surgery, received a diagnosis of FCDII, and positive genetic testing of DNA obtained from brain tissue (for more details see Table 1 and [40, 47]). Their mean age at seizure onset was 3.47 years [0–17.5; median 1.75 years], and the mean disease duration until surgery was 10.8 years [0.4–32.5; median 7 years]. Clinical data were retrieved from hospital archives. Presurgical MRI findings were reviewed by experienced neurologists and classified into (a) ‘negative’, (b) ‘subtle’, e.g., T1/T2/FLAIR signal intensity changes, blurred grey/white matter junction, with or without alterations in gyral patterning, (c) ‘distinct’ with the additional recognition of a transmantle sign or (d) ‘hemispheric’ lesions with involvement of more than one lobe. The postsurgical outcome was classified according to Engel [23]. The University of Erlangen ethical review board approved the study under agreement number 193_18B.

**Table 1:** Individuals included in the study

ID	Age	Onset	Diagnosis	Gene variant/Ref.	VAF	Family history	FU
1	33	0.5	FCD IIa	DEPDC5 (SNV) [47]	Germline	None	IVA (48)
2	20	4.5	FCD IIa	DEPDC5 (SNV) [2]	Germline	None	IIA (24)
3	42	5.5	FCD IIa	DEPDC5 (CNN-LOH) [37]	Somatic (3.7%)	Father’s sister	IIB (24)
4	12	2.8	FCD IIa	DEPDC5 (SNV + LOH) [37]	Two-hit*	None	IIA (48)
5	36	9	FCD IIa	DEPDC5 (SNV) [37]	Germline	Brother son	IA (24)
6	3	0.25	FCD IIa	DEPDC5 (CNN-LOH) [37]	Somatic (4.3%)	None	IA (24)
7	1.5	0.4	FCD IIa	DEPDC5 (SNV)^§^	Germline	Sister	IA (24)
8	5	0.7	FCD IIa	NPRL3 (SNV) [37]	Germline	None	IA (24)
9	0.4	0	FCD IIa	NPRL3 (SNV) [37]	Germline	None	IA (16)
10	17	3.5	FCD IIa	NPRL3 (SNV) [47]	Somatic (4.5%)	None	IA (24)
11	0.5	0.1	FCD Iib/HME	MTOR (SNV) [37]	Somatic (10.5%)	None	IA (24)
12	40	17.5	FCD IIa	MTOR (SNV) [37]	Somatic (5%)	None	IA (24)
13	0.8	0.3	FCD IIa/HME	MTOR (SNV) [37]	Somatic (3.4%)	None	IA (24)
14	10	3	FCD IIb	MTOR (SNV) [37]	Somatic (5.3%)	None	IA (24)
15	19	9.5	FCD IIb	MTOR (SNV) [37]	Somatic (3.5%)	None	IA (24)
16	0.9	0.2	FCD IIb	MTOR (SNV) [37]	Somatic (3.1%)	None	IA (24)
17	0.6	0	FCD IIb/PMG	MTOR (SNV) [37]	Somatic (4.3%)	None	IA (24)

Age at surgery in years; Onset: age at seizure onset; sex not shown with 50% males versus 50% females for FCD IIa and FCD IIb, respectively; Lobe: Epileptogenic focus (resulting from EEG, MRI and clinical evaluation)

*F* frontal, *T* temporal, *P* parietal, *mult* multiple lobes, *O* occipital; *Gene variant* results obtained from genetic testing, *SNV* single nucleotide variant, *CNN-LOH* copy number neutral loss of heterozygosity, *VAF* variant allelic frequency, *Ref.* specific mutation described in cited reference

*Germline variant at VAF = 43.1% and second hit somatic loss of heterozygosity at VAF = 3.7%

^$^Variant not previously published and specified below; *Diagnosis* histopathology diagnosis, *HME* hemimegalencephaly, *PMG* polymicrogyria, *FU* Follow-up 2 years after surgery according to Engel`s classification: *IA* completely seizure free, *IIA* initially seizure-free, rare seizures now, *IIB* rare seizures since surgery, *IVA* no worthwhile improvement (latest available FU in month after surgery)

### Histopathological analysis

Native surgical specimens were sectioned into 5 mm thick slabs. Representative slab(s) were fresh frozen in liquid nitrogen and stored at − 80 °C until further use [8]. The remaining tissue slabs were formalin-fixed overnight and paraffin-embedded (FFPE). Four µm thin FFPE sections were cut for each block and stained with haematoxylin–eosin. Selected blocks were further processed for Bielschowski silver staining and/or immunohistochemistry including antibodies directed against: Neurofilament-non phosphorylated (NF-SMI; clone SMI32, mouse, BioLegend, dilution 1:500), Phospho-S6 Ribosomal Protein (Ser235/236) (pS6, rabbit, Cell Signaling, dilution 1:1000), Vimentin (Vim, rabbit, Thermo, dilution 1:1000), Neuronal Nuclei (NeuN, clone A60, mouse, Millipore, dilution 1:500), Ubiquitin-binding p62/ Anti-Sequestosome 1 (p62/SQSTM1, clone P0067, rabbit, Sigma Aldrich, dilution 1:100). Immunofluorescence stainings were also performed with the p62-antibody (dilution 1:1000) and NF-SMI (dilution 1:1000) and further studies using confocal laser scanning microscopy (LSM 780, ZEISS, Germany).

Immunohistochemical stainings were performed on the Ventana BenchMark ULTRA Immunostainer using the OptiView Universal DAB Detection Kit (Ventana Medical Systems, Tucson, AZ, USA). All slides were digitalized by the Hamamatsu Nanozoomer S60. Further digital analysis was performed with QuPath v.0.3.0. We established a quantitative cell analysis for dysmorphic neurons (DN) via a single-pixel classifier. This classifier was trained individually for every sample with six representative dysmorphic neurons each and applied to two 1 mm^2^ tiles in the region of interest. DN were counted automatically as classifier-created objects, with a minimum size of 400 µm^2^ imposed. Balloon-cell quantification had to be done manually, as they were not susceptible to the pixel classifier. Here we used Vimentin staining and analysed three 1 mm^2^ tiles, respectively. In addition, we have reviewed our case series with reference to displaced dysmorphic neurons remote from the focal abnormality (defined as > 5 mm), which was graded into three levels: + = single DN > 5 mm remote from the lesion, ++ = small clusters of DN > 5 mm remote from the lesion; +++ = abundant DN > 5 mm and in continuity to the core lesion. The remote DN were identified by pS6- and SMI32 immunoreactivity.

### Genetic analysis

Whole exome sequencing was performed in DNA obtained from 13 individuals as described by Lopez-Rivera and coworker [37] (see Table 1). Variants detected in surgical brain tissue of two other individuals were previously described by Niestroj and coworker [47]. One *DEPDC5* germline variants was previously described by Baldassari and coworker [2]. The *DEPDC5* variant of individual #7 was also identified from blood cell derived DNA.

## Results

### Genetic analysis

All 17 individuals showed pathogenic variants related to the mTOR signalling pathway, i.e., *DEPDC5* (n = 7) and *NPRL3* (n = 3) associated with the GATOR1 complex, and *MTOR* (n = 7, Table 1). The mean variant allele frequency was 0.4329 for germline variants [± 0.0162] and 0.0467 for somatic variants [± 0.0194]. Among the GATOR1c variants, seven individuals had germline variants, two of which had a family history, and four individuals had somatic variants (one of them revealing both). All *MTOR* variants were brain mosaicism.

### Histopathology

**Fig. 1:**
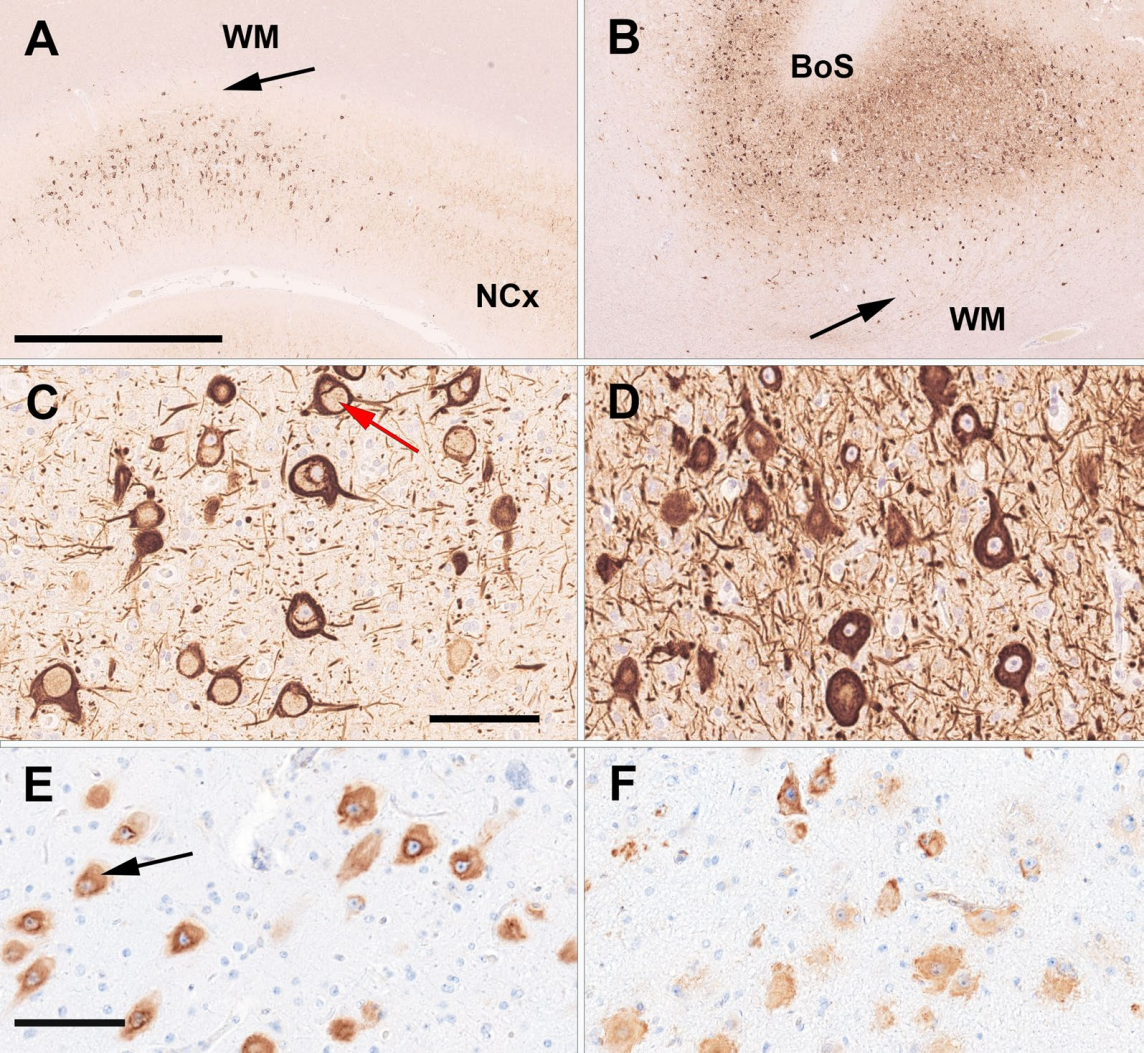
Histopathology findings of FCD ILAE Type IIa and IIb. **A** 42-year-old male individual with frontal lobe epilepsy since age 5 years (ID3). The MRI was suspicious for a bottom-of-sulcus FCD with transmantel sign. Histopathology confirmed, however, FCDIIa and a pathogenic *DEPDC5* mosaicism. The arrow points to the sharp border between the cortical FCDIIa and the normal-appearing white matter (WM). NCx—adjacent normal 6-layered neocortex. Neurofilament SMI32 immunohistochemistry. Scale bar = 2.5mm (applies also to **B**). Higher magnification in **C** reveals dysmorphic neurons with a predominant vacuolizing phenotype (red arrow) suggesting accumulation of lipofuscins and an altered autophagy pathway. Scale bar = 100 µm (applies also to **D**). Same neurons were also labeled with antibodies directed against the phospho-S6-Ser236 epitope (**E**). The black arrow in (**E**) points to a neuron with a vacuolizing phenotype. Scale bar in **E** = 100 µm, applies also to (**F**); **B** 19-year-old male individual with frontal lobe epilepsy since age nine years (ID15), histopathological confirmed FCDIIb at a bottom-of-sulcus (BoS; higher magnification in **D**) and a pathogenic *MTOR* mosaicism. Dysmorphic neurons and balloon cells were aggregated in the neocortex and white matter (arrow in **B**) compatible with a migration-deficient phenotype. **F** both, dysmorphic neurons and balloon cells were labelled with antibodies directed against the pS6 Ser236 epitope

In all cases, a systematic histopathological analysis revealed an association between GATOR1 variants *DEPDC5* and *NPRL3* with FCD ILAE type IIa (Table 1). In contrast, five specimens with *MTOR* variants also revealed balloon cells, defining them as FCD ILAE type IIb. Three individuals with *MTOR* brain mosaicism had additional pathologies (HME n = 2, PMG n = 1). pS6 immunoreactivity was performed in all tissue specimens and confirmed activation of mTOR signalling in affected dysmorphic neurons and/or balloon cells (Fig. 1E/F). All FCDIIa lesions affected only the neocortex without spread into adjacent white matter (Fig. 1A). This observation was in contrast to MTOR-associated FCDIIb, with all lesions affecting the neocortex and the white matter (Fig. 1B).

**Fig. 2:**
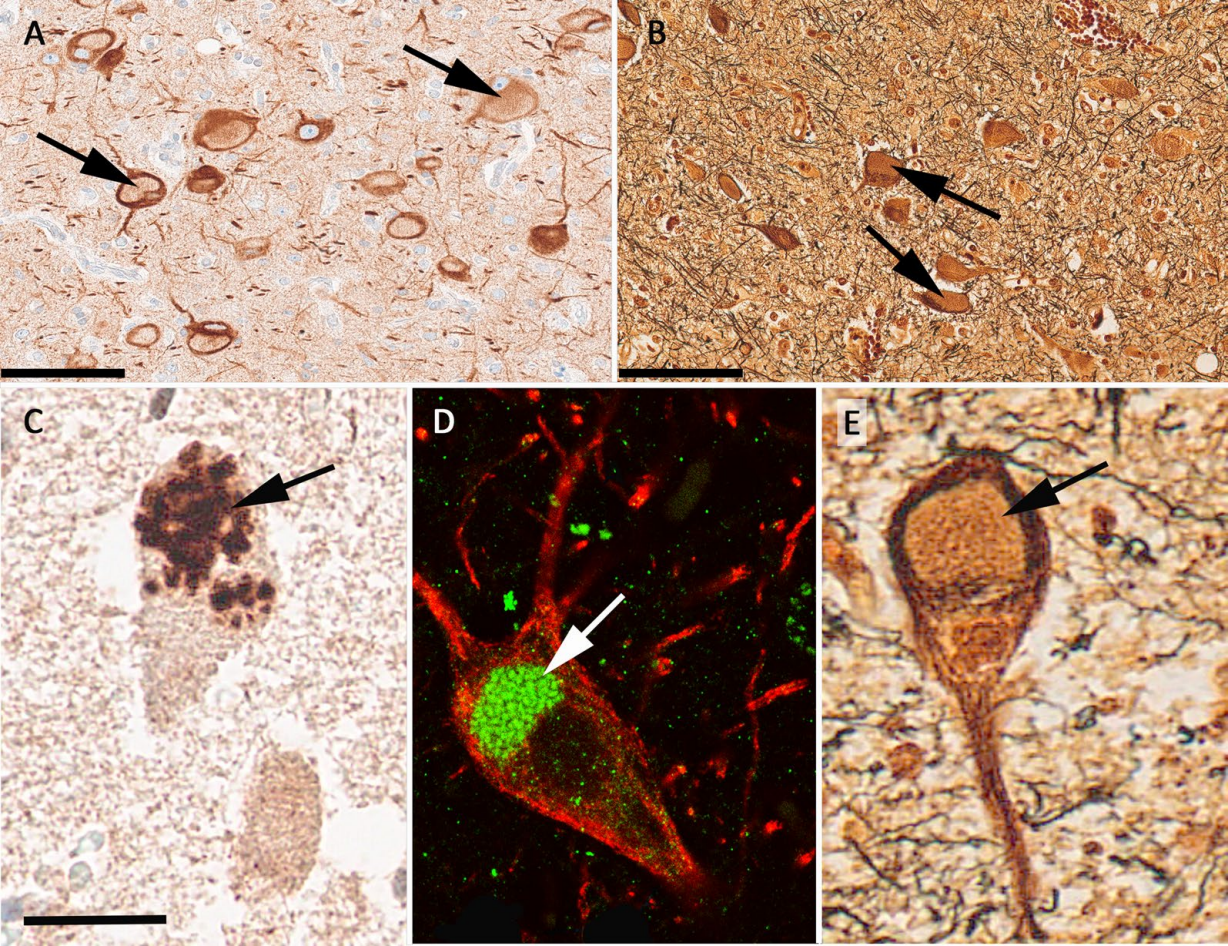
Autophagocytic phenotype in *DEPDC5* altered FCDIIa. Images were taken from a 42-year-old male individual with drug-resistant focal epilepsy and FCDIIa, *DEPDC5* altered (ID3). A juxtanuclear accumulation of autophagosomes can be anticipated from the displacement of neurofilaments (arrow in **A** as an example, NF-SMI32) and silver impregnation (arrow in **B**, Bielschowski silver staining, see also higher magnification in **E**). **C** p62-immunoreactivity highlighted the aggregation of autophagosomes in FCDIIa. **D** Further confirmation of the autophagocytic phenotype in FCDIIa by double immunofluorescence and laser scanning microscopy of NF-SMI32 (in red) and p62 (in green). Scale bar in **A** and **B** = 100 µm. The scale bar in **C** = 25 µm, applies also to (**D**) and (**E**)

Automatized measurement of the density of dysmorphic neurons did not show any significant difference between the genetic subgroups associated with GATOR1c or MTOR (*p* = 0.0876). A correlation between VAF and the density of either balloon cells or dysmorphic neurons was also not seen. Five out of 10 cases with GATOR-complex variants had a unique vacuolizing predominant phenotype recognized in neurofilament stainings (NF-SMI32), which could not be detected in any case with *MTOR* variants (Fig. 2). This was confirmed by a distinct vesicular p62-immunoreactivity pattern (Fig. 2D). Such juxtanuclear p62 accumulation was not recognizable in any sample of FCDIIb carrying *MTOR* variants. In addition, the cytopathology was mainly localized to the neocortex, i.e., a focal abnormality. This did not exclude the presence of individual pS6- and SMI32-immunoreactive dysmorphic neurons in areas remote from the focal abnormality irrespective from the affected genetic variant, VAF, or germline mutation (Table 2). However, 70% of GATOR positive compared to 28% of MTOR positive FCDII only had single remote DN.

**Table 2:** Correlation of neuropathology with neuroimaging findings

ID	MRI localization	MRI class	MRI alterations in GM and/or WM	Histopath of WM	Balloon cells	vacuolizing phenotype	Remote DN	DN density
1	Left F	Subtle	T2 blurred GM/WM junction with cortical dimple	−	−	+	+	67 [± 0]
2	Right F	Negative	None visible	−	−	−	+	NA
3	Right F	Distinct	FLAIR blurred GM/WM junction, TMS	−	−	+	+	42.5 [± 3.5]
4	Right F	Negative	None visible	−	−	+	+	58 [± 8]
5	Right F	Subtle	FLAIR blurred GM/WM junction, T1 cortex hyperintensity, no TMS	−	−	+	+	43.5 [± 11.5]
6	Left F	Subtle	Cortical thickening, GM/WM T2 hypointense and T1 hyperintense at 3 months, no TMS	−	−	−	++	96.5 [± 9.5]
7	Left F	Distinct	Cortical thickening, GM/WM T2 hypointense and T1 hyperintense at 10 months, no TMS	−	−	−	++	89 [± 3]
8	Left TP	Subtle	FLAIR cortical thickening, FLAIR blurred GM/WM junction, no TMS	−	−	−	+	NA
9	Right FI	Hem	HME—cortical thickening, GM/WM T2 hypo-/T1-hyperintense at 5 months, no TMS	−	−	−	++	42.5 [± 8.5]
10	Left F	Subtle	T2 + FLAIR cortical thickening T2 + FLAIR blurred GM/WM junction, no TMS	−	−	+	+	95.5 [± 12.5]
11	Right FPO	Hem	HME—cortical thickening, GM/WM T2 hypointense at 3 months, no TMS	+	+	−	+++	37 [± 2]
12	Left F	Uncertain	MRI of low quality, pacemaker implantation	+	−	−	++	27 [± 5]
13	Right H	Hem	HME—cortical thickening, GM/WM T2 hypointense at 3 months, no TMS	−	−	−	++	14.5 [± 4.5]
14	Left F	Distinct	GM/WM FLAIR hyperintense, T1 hypointense, TMS	+	+	−	+++	33 [± 4]
15	Right F	Distinct	FLAIR blurred GM/WM junction, TMS	+	+	−	+	73.5 [± 12.5]
16	Left PO	Distinct	Thickened cortex, GM/WM T2 hyperintense, T1 isointense at 7 months, no TMS	−	+	−	+	44 [± 1]
17	Right F	Distinct	Cortical thickening w sulcal dimple, GM/WM T2 hypointense at 5 months, no TMS	+	+	−	+++	70 [± 17]

LID same as in Table 1; MRI findings: All images from MRI datasets were reviewed and classified as “distinct”, e.g., thickened neocortex with distinct signal intensity change in T1, T2 and/or FLAIR with or without transmantle sign (TMS), or “subtle”, e.g. grey/white matter (GM/WM) blurring and/or altered gyration patterns, *hem* hemispheric, *HME* hemimegalencephaly, *PMG* polymicrogyria, *WM* white matter affection, Balloon cells and vacuolizing phenotype: + positive, − negative, *DN density* dysmorphic neurons per mm^2^ as mean ± standard deviation, *NA* surgical specimen fragmented and not available for analysis; *Remote DN* dysmorphic neurons remote from the focal abnormality (> 5 mm) graduated in three different levels: + = single DN > 5 mm remote from the lesion, ++ = small clusters of DN > 5 mm remote from the lesion; +++ = abundant DN > 5 mm in continuity to the core lesion; *MRI Localization* localization of lesion on MRI with side and lobe(s), *F* frontal, *P* parietal, *O* occipital, T temporal, *H* hemisphere, *FLAIR* fluid attenuated inversion recovery, *T1* T1 weighted image, *T2* T2 weighted image

### Neuroimaging analysis and postsurgical outcome

**Fig. 3:**
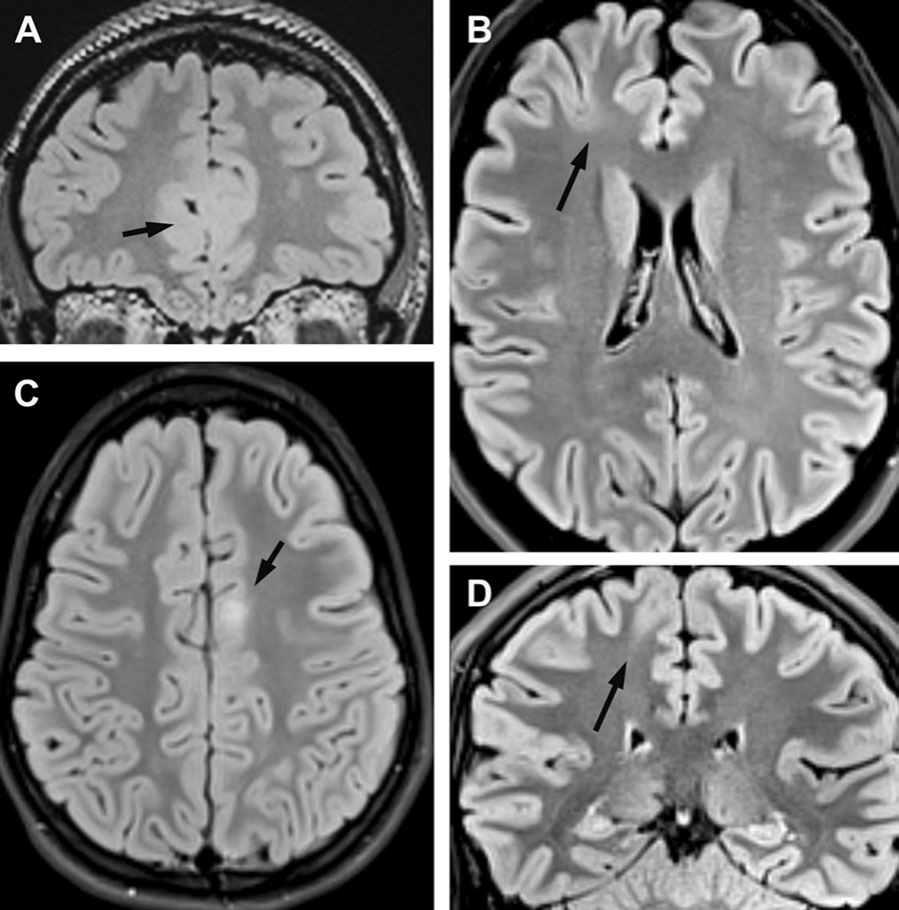
Representative FLAIR neuroimaging findings of FCDIIa, DEPDC5 altered and FCDIIb, MTOR altered. **A** individual ID4 with histopathologically confirmed FCDIIa and a two-hit *DEPDC5* variant. The MRI showed no definite lesion and the right fronto-mesial lobe was surgically removed (arrow); **B** individual ID5, classified as bottom-of-sulcus FCD; **C** individual ID14 with a thickened neocortex and distinct signal intensity change in FLAIR; **D** individual ID15 with a distinct transmantle sign; Individual #14 and #15 both revealed somatic *MTOR* variants

Thirteen individuals were seizure-free after surgical treatment (76.5% Engel IA; Table 1). All five individuals not being seizure free shared the same histopathology diagnosis of FCDIIa and carried *DEPDC5* variants (Table 1). Two individuals with DEPDC5 germline variants also had a positive family history of epilepsy. There was no significant difference between the histopathologically defined FCDIIa and FCDIIb subtypes concerning age at seizure onset or disease duration. However, we recognized a higher age of GATOR positive patients with vacuolated cells (i.e. 12, 17, 33, 36 and 42 years old at surgery) compared to GATOR positive patients not showing vacuolated neurons (age 0.4, 5, 1.5, 3, 20). Importantly, presurgical MRI findings were subtle in five and negative in two individuals with FCDIIa compared to distinct lesions in four and a hemispheric lesion of individuals with FCDIIb (Fig. 3). All individuals with *DEPDC5* variants had their neurosurgical resection confined to the frontal lobe, while three individuals with *MTOR* variants had multilobar lesions and frontal involvement in six of seven cases. There was no difference in the hemispheric side of FCDIIa or FCDIIb.

## Discussion

Our study revealed a hitherto under-recognized genotype–phenotype association for FCDIIa with (1) GATOR1 complex variants in the frontal lobe, i.e., *DEPDC5* and *NPRL3*, (2) subtle MRI visibility of the lesion, and (3) the lesion restricted to the neocortex at the microscopy level and autophagosome accumulation in dysmorphic neurons. Importantly, four of ten individuals with FCDIIa were not seizure-free following surgical treatment. This contrasted the genotype–phenotype association of FCDIIb, as all individuals carried a brain somatic *MTOR* variant, had distinct and clearly visible MRI lesions, and were seizure-free after surgery. At the microscopy level, *MTOR*-positive FCDIIb revealed a migration-deficient phenotype retaining dysmorphic neurons and balloon cells in the white matter.

Our current literature search for studies reporting the genotype of histopathologically well-characterized FCD ILAE Type II lesions identified ten articles (Table 3). We can readily anticipate from these studies a predominant association of 77% of FCDIIb with mTORC1 alterations, whereas GATOR1 alterations were detected in 67% of all genetically positive FCDIIa. In our case series, however, all GATOR1-positive lesions were histopathologically characterized by FCDIIa, whereas two other FCDIIa lesions were associated with *MTOR* variants (Table 1). This observation is in line with five of the seven published series reporting genetically confirmed FCDIIa (Table 3), raising this association to 22 of 23 cases (96%). Notwithstanding, it also reiterates the issue of small sample numbers in such case studies and the difficulty of reliably classifying the FCD subtype at the histopathology level [10, 16, 18].

**Table 3:** Previously published FCDII lesions with a positive genetic finding and histopathologically proven using the 2011 ILAE classification scheme

References	# of FCDII^genetic+^ of all individuals	FCDIIa^GATOR1+^ of all FCD IIa	FCDIIa^MTORC1^ of all FCD IIa	FCDIIb^MTORC1^ of all FCD IIb	FCDIIb^GATOR1+^ of all FCD IIb
Lim et al. [37]	12/12	–	5/12	7/12	–
Nakashima et al. [45]	6/6	–	–	6/6	–
Baulac et al. [4]	2/4	2/2	–	–	–
D’Gama et al. [21]	5/18	2/2	–	2/3	–
Ying et al. [63]	1/10	1/1	–	–	–
Niestroj et al. [47]	4/15	2/3	–	1/1	–
Baldassari et al. [2]	34/43	5/19	10/19	9/15	–
Blümcke et al. [10]	4/22	1/3	1/3	1/1	–
Wang et al. [61]	10/20	–	–	10/10	–
Wang et al. [60]	15/50	15/15	–	–	–
This study	17/17	10/12	2/12	5/5	–
Total	110/217	38/57 (67%)	18/46(39%)	41/53 (77%)	–

*#* *of FCDII*^*genetic*+^ *of all individuals* number of histopathologically confirmed FCDII with a genetic lesion/of all reported individuals, *FCDIIa*^*GATOR1*+^ FCD ILAE Type IIa with a variant directly affecting the GATOR1 complex, e.g. *DEPDC5* or *NPRL3*/of all reported FCDIIa^genetic+^ in that study, *FCDIIb*^*MTORC1*^ FCD ILAE Type IIb with a variant in *MTORC1* activating genes of all reported FCDIIb^genetic+^ in that study, *FCDIIb*^*GATOR1*+^ FCD ILAE Type IIb with a variant in *GATOR1 complex* of all reported FCDIIb^genetic+^ in that study, *FCDIIa*^*MTORC1*^ FCD ILAE Type IIa with a variant in *MTOR* complex 1 activating genes of all reported FCDIIa^genetic+^ in that study*.* The publication of Lopez-Rivera et al. [40] was excluded herein as it included same cases used in the present study

Interestingly, a previous case study described dysmorphic neurons with significant lipofuscin accumulation as a new disease entity in six individuals, i.e., focal neuronal lipofuscinosis (NFL), distinct from FCD Type IIa or IIb [38], as confirmed by a recent and independent case report [43]. However, these studies did not include any genetic analysis. At the same time the clinico-pathological similarity to our individual series is overwhelming [38]: (1) loss of DEPDC5 immunoreactivity in the population of dysmorphic neurons, (2) activation of the autophagy pathway, (3) lesions confined to the neocortex not involving the white matter, and (4) subtle MRI findings, respectively. Their observation prompted them to conclude a new disease entity separate from FCD ILAE Type II, when compared to six individuals with FCDIIb or seven individuals without any histopathology findings used as control. Although we cannot exclude that p62-immunoreactive aggregates include other material than just lipofuscins or that the Ser236 antibody used herein may have different labelling properties compared to the Ser240 epitope, we suggest our five cases and NFL being the same disease to be classified according to the updated FCD classification scheme of 2022 [44] as: MRI-positive *DEPDC5*- or *NPRL3*-altered Focal Cortical Dysplasia ILAE Type IIa.

Loss-of-function variants of the GATOR1 subunits DEPDC5, NPRL2, and NPRL3 in FCDIIa result in constitutive activation of mTORC1 [3, 4, 58] and are likely similar to that mediated by activating *MTOR* variants. Yet, we cannot explain the predominant autophagocytic phenotype in dysmorphic neurons of FCDIIa and could not directly measure an up or down regulation of autophagy in the FFPE tissue samples [25]. However, evidence for a change in autophagy rate was provided by the study proposing the NLF phenotype [38].

Autophagy and neuronal migration dysregulations can be assigned to the portfolio of the mTOR signalling pathway [30, 37]. In contrast to GATOR1 complex variants, individuals with FCDIIb and brain somatic *MTOR* variants showed larger lesions, including the white matter in histopathology, which was often reflected also by MRI findings [61]. This suggested a stronger pathogenic focus on neuronal migration in the latter group. Experimental evidence showed that activation of mTORC1 leads to impaired cortical lamination of cytomegalic neurons and cortical hyperplasia in adult animals [19, 21, 31]. Intriguingly, a migration defect was primarily attenuated in our FCDIIa^GATOR1+^ group. These phenotypic differences associated with *MTOR* or GATOR1 variants implicate divergent mechanisms during cortical development despite convergent mTORC1 hyperactivation. The timing of the acquired gene alteration and the targeted cell population may explain these differences, as previously shown in experimental animal models [10, 21, 37, 46]. In addition a *DEPDC5* two-hit model may be needed to cause FCDIIa [2, 4, 36, 40, 54, 56], as observed in this study's carrier of a germline/somatic DEPDC5 double-hit variant.

The predictability of postsurgical seizure freedom is of great concern for individual management and often guides the decision-making process [29]. MRI visibility of a circumscript lesion and its surgical accessibility are regarded as key factors for good outcome, however. Interestingly, the limitation of FCDIIa, GATOR1-altered lesions, to the neocortex represented a histopathological correlate for subtle or negative MRI findings in our small case series, e.g., lack of a transmantle sign (Fig. 3). This may help to understand another observation in the landscape of epilepsy-related GATOR1 variants, where the most frequent entities are focal epilepsies and often present with MRI-negative/-subtle findings [4]. We also reviewed the published literature on postsurgical outcome in genetically proven FCDII and identified a recent review of 8 children with GATOR positive drug resistant focal epilepsy, of which 4 children did not become seizure free [55]. Their further literature review of GATOR positive cases, where the most encountered pathology was FCDIIa, indicated an overall seizure freedom rate of 60%, a number very similar to ours reported in the current study [55]. Although there was no evidence of any residual dysplasia on postoperative MRI in our cases, that were not seizure free, glial scarring at the resection borders may have obscured such findings.

Genotype–phenotype associations come of age in neuropathology, foremost diagnosing brain tumours, as controversies in microscopic agreement compromised the liability of the histopathology report for decades [7, 11, 52]. Insights into the molecular pathogenesis to better understand the underlying cause, disease prognosis, or targeted treatment options further promoted the integration of molecular neuropathology into clinical practice [41, 42]. A classic example is that of mixed oligo-astrocytoma which became obsolete in the 5th edition of the WHO brain tumour classification scheme [42]. The international consensus FCD classification update 2022 also introduced an integrated classification scheme of histopathological and molecular layers [6, 44]. Such integrated diagnosis will improve our understanding of FCD subtypes and their clinical management and help develop targeted treatment options. Increasing the availability and access to smart drugs targeting mTOR-associated genes will further strengthen the ILAE approach to classify FCD at an integrated molecular pathology level. However, the distinction between FCDIIa and FCDIIb remained often academic as the presence or absence of balloon cells can be a subjective measure when trying to differentiate reactive, gemistocytic astrocytes in areas targeted by intracerebral EEG recordings or from surgical sampling errors in small lesions or functional hemispherotomy. Interrater disagreement due to lack of training facilities or access to specific laboratory resources and protocols may represent another obstacle, e.g., an immunohistochemistry panel for epilepsy [8, 10]. The current individual cohort took all available precaution to rule out such bias and revealed a robust genotype–phenotype association impacting surgical outcomes.

In conclusion, our study revealed phenotypic and genotypic signatures in FCD subtype IIa and IIb despite a close relationship of affected mTOR pathway genes, i.e., *DEPDC5*, *NPRL3,* and *MTOR*, as well as converging histopathology findings, e.g., cortical dyslamination and dysmorphic neurons. Their association with MRI visibility or adverse surgical outcome will gain attention. It will have consequences in the search for precision medicine tools, e.g., presurgical germline testing for GATOR1-associated candidate genes, as more personalized treatment options will become available. This strategy aligns with contemporary disease classification schemes in histopathology integrating the clinical, microscopic and molecular level to help better understand difficult-to-diagnose FCD.

### Supplementary Information

**Additional file 1.** Genetic findings with detailed variant descriptions.

## Data Availability

All data generated or analysed during this study are included in this published article and its Additional file 1.

## References

[1] Andrews MG, Subramanian L, Kriegstein AR (2020). mTOR signaling regulates the morphology and migration of outer radial glia in developing human cortex. Elife.

[2] Baldassari S, Ribierre T, Marsan E, Adle-Biassette H, Ferrand-Sorbets S, Bulteau C, Dorison N, Fohlen M, Polivka M, Weckhuysen S (2019). Dissecting the genetic basis of focal cortical dysplasia: a large cohort study. Acta Neuropathol.

[3] Bar-Peled L, Chantranupong L, Cherniack AD, Chen WW, Ottina KA, Grabiner BC, Spear ED, Carter SL, Meyerson M, Sabatini DM (2013). A tumor suppressor complex with GAP activity for the Rag GTPases that signal amino acid sufficiency to mTORC1. Science.

[4] Baulac S, Ishida S, Marsan E, Miquel C, Biraben A, Nguyen DK, Nordli D, Cossette P, Nguyen S, Lambrecq V (2015). Familial focal epilepsy with focal cortical dysplasia due to DEPDC5 variants. Ann Neurol.

[5] Bernasconi A, Cendes F, Theodore WH, Gill RS, Koepp MJ, Hogan RE, Jackson GD, Federico P, Labate A, Vaudano AE (2019). Recommendations for the use of structural magnetic resonance imaging in the care of patients with epilepsy: a consensus report from the International League Against Epilepsy Neuroimaging Task Force. Epilepsia.

[6] Blumcke I (2019). It is time to move on: commentary to: genotype–phenotype correlations in focal malformations of cortical development: a pathway to integrated pathological diagnosis in epilepsy surgery. Brain Pathol.

[7] Blümcke I, Aronica E, Becker A, Capper D, Coras R, Honavar M, Jacques TS, Kobow K, Miyata H, Mühlebner A (2016). Low-grade epilepsy-associated neuroepithelial tumours—the 2016 WHO classification. Nat Rev Neurol.

[8] Blumcke I, Aronica E, Miyata H, Sarnat HB, Thom M, Roessler K, Rydenhag B, Jehi L, Krsek P, Wiebe S (2016). International recommendation for a comprehensive neuropathologic workup of epilepsy surgery brain tissue: a consensus Task Force report from the ILAE Commission on Diagnostic Methods. Epilepsia.

[9] Blumcke I, Budday S, Poduri A, Lal D, Kobow K, Baulac S (2021). Neocortical development and epilepsy: insights from focal cortical dysplasia and brain tumours. Lancet Neurol.

[10] Blumcke I, Coras R, Busch RM, Morita-Sherman M, Lal D, Prayson R, Cendes F, Lopes-Cendes I, Rogerio F, Almeida VS (2021). Toward a better definition of focal cortical dysplasia: an iterative histopathological and genetic agreement trial. Epilepsia.

[11] Blumcke I, Coras R, Wefers AK, Capper D, Aronica E, Becker A, Honavar M, Stone TJ, Jacques TS, Miyata H (2019). Challenges in the histopathological classification of ganglioglioma and DNT: microscopic agreement studies and a preliminary genotype-phenotype analysis. Neuropathol Appl Neurobiol.

[12] Blümcke I, Spreafico R, Haaker G, Coras R, Kobow K, Bien CG, Pfafflin M, Elger C, Widman G, Schramm J (2017). Histopathological findings in brain tissue obtained during epilepsy surgery. N Engl J Med.

[13] Blümcke I, Thom M, Aronica E, Armstrong DD, Vinters HV, Palmini A, Jacques TS, Avanzini G, Barkovich AJ, Battaglia G (2011). The clinico-pathological spectrum of focal cortical dysplasias: a consensus classification proposed by an ad hoc Task Force of the ILAE Diagnostic Methods Commission. Epilepsia.

[14] Boonyapisit K, Najm I, Klem G, Ying Z, Burrier C, LaPresto E, Nair D, Bingaman W, Prayson R, Luders H (2003). Epileptogenicity of focal malformations due to abnormal cortical development: direct electrocorticographic-histopathologic correlations. Epilepsia.

[15] Cepeda C, Hurst RS, Flores-Hernandez J, Hernandez-Echeagaray E, Klapstein GJ, Boylan MK, Calvert CR, Jocoy EL, Nguyen OK, Andre VM (2003). Morphological and electrophysiological characterization of abnormal cell types in pediatric cortical dysplasia. J Neurosci Res.

[16] Chamberlain WA, Cohen ML, Gyure KA, Kleinschmidt-DeMasters BK, Perry A, Powell SZ, Qian J, Staugaitis SM, Prayson RA (2009). Interobserver and intraobserver reproducibility in focal cortical dysplasia (malformations of cortical development). Epilepsia.

[17] Chung C, Yang X, Bae T, Vong KI, Mittal S, Donkels C, Westley Phillips H, Li Z, Marsh APL, Breuss MW (2023). Comprehensive multi-omic profiling of somatic variants in malformations of cortical development. Nat Genet.

[18] Coras R, de Boer OJ, Armstrong D, Becker A, Jacques TS, Miyata H, Thom M, Vinters HV, Spreafico R, Oz B (2012). Good interobserver and intraobserver agreement in the evaluation of the new ILAE classification of focal cortical dysplasias. Epilepsia.

[19] Crino PB (2016). The mTOR signalling cascade: paving new roads to cure neurological disease. Nat Rev Neurol.

[20] D'Gama AM, Geng Y, Couto JA, Martin B, Boyle EA, LaCoursiere CM, Hossain A, Hatem NE, Barry BJ, Kwiatkowski DJ (2015). Mammalian target of rapamycin pathway variants cause hemimegalencephaly and focal cortical dysplasia. Ann Neurol.

[21] D'Gama AM, Woodworth MB, Hossain AA, Bizzotto S, Hatem NE, LaCoursiere CM, Najm I, Ying Z, Yang E, Barkovich AJ (2017). Somatic variants activating the mTOR pathway in dorsal telencephalic progenitors cause a continuum of cortical dysplasias. Cell Rep.

[22] Dibbens LM, de Vries B, Donatello S, Heron SE, Hodgson BL, Chintawar S, Crompton DE, Hughes JN, Bellows ST, Klein KM (2013). Variants in DEPDC5 cause familial focal epilepsy with variable foci. Nat Genet.

[23] Engel JJ, Van Ness P, Rasmussen TB, Ojemann LM, Engel JJ (1993). Outcome with respect to epileptic seizures. Surgical treatment of the epilepsies.

[24] Gerasimenko A, Baldassari S, Baulac S (2023). mTOR pathway: insights into an established pathway for brain mosaicism in epilepsy. Neurobiol Dis.

[25] Hui KK, Tanaka M (2019). Autophagy links MTOR and GABA signaling in the brain. Autophagy.

[26] Iffland PH, Carson V, Bordey A, Crino PB (2019). GATORopathies: the role of amino acid regulatory gene variants in epilepsy and cortical malformations. Epilepsia.

[27] Iffland PH, Crino PB (2017). Focal cortical dysplasia: gene variants, cell signaling, and therapeutic implications. Annu Rev Pathol.

[28] Inoki K, Zhu T, Guan KL (2003). TSC2 mediates cellular energy response to control cell growth and survival. Cell.

[29] Jehi L, Braun K (2021). Does etiology really matter for epilepsy surgery outcome?. Brain Pathol.

[30] Jung CH, Ro SH, Cao J, Otto NM, Kim DH (2010). mTOR regulation of autophagy. FEBS Lett.

[31] Kassai H, Sugaya Y, Noda S, Nakao K, Maeda T, Kano M, Aiba A (2014). Selective activation of mTORC1 signaling recapitulates microcephaly, tuberous sclerosis, and neurodegenerative diseases. Cell Rep.

[32] Kim DH, Sarbassov DD, Ali SM, King JE, Latek RR, Erdjument-Bromage H, Tempst P, Sabatini DM (2002). mTOR interacts with raptor to form a nutrient-sensitive complex that signals to the cell growth machinery. Cell.

[33] Lai D, Gade M, Yang E, Koh HY, Lu J, Walley NM, Buckley AF, Sands TT, Akman CI, Mikati MA (2022). Somatic variants in diverse genes leads to a spectrum of focal cortical malformations. Brain.

[34] Lee JH, Huynh M, Silhavy JL, Kim S, Dixon-Salazar T, Heiberg A, Scott E, Bafna V, Hill KJ, Collazo A (2012). De novo somatic variants in components of the PI3K-AKT3-mTOR pathway cause hemimegalencephaly. Nat Genet.

[35] Lee WS, Leventer RJ, Lockhart PJ (2022). Droplet digital PCR as a first-tier molecular diagnostic tool for focal cortical dysplasia type II. Brain.

[36] Lee WS, Stephenson SEM, Howell KB, Pope K, Gillies G, Wray A, Maixner W, Mandelstam SA, Berkovic SF, Scheffer IE (2019). Second-hit DEPDC5 variant is limited to dysmorphic neurons in cortical dysplasia type IIA. Ann Clin Transl Neurol.

[37] Lim JS, Kim WI, Kang HC, Kim SH, Park AH, Park EK, Cho YW, Kim S, Kim HM, Kim JA (2015). Brain somatic variants in MTOR cause focal cortical dysplasia type II leading to intractable epilepsy. Nat Med.

[38] Liu JY, Reeves C, Diehl B, Coppola A, Al-Hajri A, Hoskote C, Mughairy SA, Tachrount M, Groves M, Michalak Z (2016). Early lipofuscin accumulation in frontal lobe epilepsy. Ann Neurol.

[39] Loewith R, Jacinto E, Wullschleger S, Lorberg A, Crespo JL, Bonenfant D, Oppliger W, Jenoe P, Hall MN (2002). Two TOR complexes, only one of which is rapamycin sensitive, have distinct roles in cell growth control. Mol Cell.

[40] Lopez-Rivera JA, Leu C, Macnee M, Khoury J, Hoffmann L, Coras R, Kobow K, Bhattarai N, Perez-Palma E, Hamer H (2022). The genomic landscape across 474 surgically accessible epileptogenic human brain lesions. Brain.

[41] Louis DN, Perry A, Reifenberger G, von Deimling A, Figarella-Branger D, Cavenee WK, Ohgaki H, Wiestler OD, Kleihues P, Ellison DW (2016). The 2016 World Health Organization classification of tumors of the central nervous system: a summary. Acta Neuropathol.

[42] Louis DN, Perry A, Wesseling P, Brat DJ, Cree IA, Figarella-Branger D, Hawkins C, Ng HK, Pfister SM, Reifenberger G (2021). The 2021 WHO classification of tumors of the central nervous system: a summary. Neuro Oncol.

[43] Mhatre R, Jagtap SA, Kurwale N, Santhoshkumar R, Deshmukh Y, Mahadevan A (2020). Frontal lobe epilepsy with focal neuronal lipofuscinosis—case report of a rare entity. Epilepsy Behav Rep.

[44] Najm I, Lal D, Vanegas MA, Cendes F, Lopes-Cendes I, Palmini A, Paglioli E, Sarnat H, Walsh CA, Wiebe S (2022). The ILAE consensus classification of focal cortical dysplasia (FCD): an update proposed by an ad hoc Task Force of the ILAE Diagnostic Methods Commission. Epilepsia.

[45] Nakashima M, Saitsu H, Takei N, Tohyama J, Kato M, Kitaura H, Shiina M, Shirozu H, Masuda H, Watanabe K (2015). Somatic variants in the MTOR gene cause focal cortical dysplasia type IIb. Ann Neurol.

[46] Nguyen LH, Bordey A (2021). Convergent and divergent mechanisms of epileptogenesis in mtoropathies. Front Neuroanat.

[47] Niestroj LM, May P, Artomov M, Kobow K, Coras R, Pérez-Palma E, Altmüller J, Thiele H, Nürnberg P, Leu C (2019). Assessment of genetic variant burden in epilepsy-associated brain lesions. Eur J Hum Genet.

[48] Palmini A, Gambardella A, Andermann F, Dubeau F, da Costa JC, Olivier A, Tampieri D, Gloor P, Quesney F, Andermann E (1995). Intrinsic epileptogenicity of human dysplastic cortex as suggested by corticography and surgical results. Ann Neurol.

[49] Palmini A, Najm I, Avanzini G, Babb T, Guerrini R, Foldvary-Schaefer N, Jackson G, Luders HO, Prayson R, Spreafico R (2004). Terminology and classification of the cortical dysplasias. Neurology.

[50] Peterson TR, Laplante M, Thoreen CC, Sancak Y, Kang SA, Kuehl WM, Gray NS, Sabatini DM (2009). DEPTOR is an mTOR inhibitor frequently overexpressed in multiple myeloma cells and required for their survival. Cell.

[51] Pirozzi F, Berkseth M, Shear R, Gonzalez L, Timms AE, Sulc J, Pao E, Oyama N, Forzano F, Conti V (2022). Profiling PI3K-AKT-MTOR variants in focal brain malformations reveals new insights for diagnostic care. Brain.

[52] Ramaswamy V, Taylor MD (2016). Fall of the optical wall: freedom from the tyranny of the microscope improves glioma risk stratification. Cancer Cell.

[53] Rampp S, Rossler K, Hamer H, Illek M, Buchfelder M, Doerfler A, Pieper T, Hartlieb T, Kudernatsch M, Koelble K (2021). Dysmorphic neurons as cellular source for phase-amplitude coupling in focal cortical dysplasia type II. Clin Neurophysiol.

[54] Ribierre T, Deleuze C, Bacq A, Baldassari S, Marsan E, Chipaux M, Muraca G, Roussel D, Navarro V, Leguern E (2018). Second-hit mosaic variant in mTORC1 repressor DEPDC5 causes focal cortical dysplasia-associated epilepsy. J Clin InvestIG.

[55] Sahly A, Whitney R, Costain G, Chau V, Otsubo H, Ochi A, Donner E, Cunningham J, Jones K, Widjaja E, Ibrahim G, Jain P (2023). Epilepsy surgery outcomes in patients with GATOR1 gene complex variants: Report of new cases and review of literature. Seizure Eur J Epilepsy.

[56] Sim NS, Ko A, Kim WK, Kim SH, Kim JS, Shim KW, Aronica E, Mijnsbergen C, Spliet WGM, Koh HY (2019). Precise detection of low-level somatic variant in resected epilepsy brain tissue. Acta Neuropathol.

[57] Tassi L, Colombo N, Garbelli R, Francione S, Lo Russo G, Mai R, Cardinale F, Cossu M, Ferrario A, Galli C (2002). Focal cortical dysplasia: neuropathological subtypes, EEG, neuroimaging and surgical outcome. Brain.

[58] van Kranenburg M, Hoogeveen-Westerveld M, Nellist M (2015). Preliminary functional assessment and classification of DEPDC5 variants associated with focal epilepsy. Hum Mutat.

[59] Von Oertzen J, Urbach H, Jungbluth S, Kurthen M, Reuber M, Fernandez G, Elger CE (2002). Standard magnetic resonance imaging is inadequate for patients with refractory focal epilepsy. J Neurol Neurosurg Psychiatry.

[60] Wang H, Liu W, Zhang Y, Liu Q, Cai L, Jiang Y (2023). Seizure features and outcomes in 50 children with GATOR1 variants: a retrospective study, more favorable for epilepsy surgery. Epilepsia Open.

[61] Wang Y, Yu T, Blumcke I, Cai Y, Sun K, Gao R, Wang Y, Fu Y, Wang W, Wang Y (2023). The clinico-pathological characterization of focal cortical dysplasia type IIb genetically defined by MTOR mosaicism. Neuropathol Appl Neurobiol.

[62] Wang ZI, Jones SE, Jaisani Z, Najm IM, Prayson RA, Burgess RC, Krishnan B, Ristic A, Wong CH, Bingaman W (2015). Voxel-based morphometric magnetic resonance imaging (MRI) postprocessing in MRI-negative epilepsies. Ann Neurol.

[63] Ying Z, Wang I, Blümcke I, Bulacio J, Alexopoulos A, Jehi L, Bingaman W, Gonzalez-Martinez J, Kobow K, Niestroj LM (2019). A comprehensive clinico-pathological and genetic evaluation of bottom-of-sulcus focal cortical dysplasia in patients with difficult-to-localize focal epilepsy. Epileptic Disord.

