# Supplementary material for: Deep histopathology genotype–phenotype analysis of focal cortical dysplasia type II differentiates between the GATOR1-altered autophagocytic subtype IIa and MTOR-altered migration deficient subtype IIb
Source: Acta Neuropathol Commun. 2023 Nov 9;11:179. doi: 10.1186/s40478-023-01675-x (PMC10633947; doi:10.1186/s40478-023-01675-x)
Supplement: Supplementary file 1 — Additional file 1. Genetic findings with detailed variant descriptions. [file 40478_2023_1675_MOESM1_ESM.docx]

**Supplement:**

**Genetic findings with detailed variant descriptions**

| **ID** | **variant** |
| --- | --- |
| **1** | germline *DEPDC5* NM_001242897.1:c.4012C>T, p. (Gln1338*) |
| **2** | germline *DEPDC5* NM_001242896.3:c.483+1G>A |
| **3** | somatic *DEPDC5* CNN-LOH: chr22:b37_start=18009909:b37_stop=49085205 |
| **4** | germline *DEPDC5*: NM_001242896:c.2620C>T, p.(Arg874*) + somatic *DEPDC5* CNN-LOH: chr22:b37_start=19183787:b37_stop=51304566 |
| **5** | germline *DEPDC5*: NM_001242896:c.715C>T, p.(Arg239*) |
| **6** | somatic *DEPDC5* CNN-LOH: chr22:b37_start=16000000:b37_stop=51304566 |
| **7** | DEPDC5 (NM_001242896.1), c.1400_1401insGG, p.(Phe467Leufs*51) |
| **8** | germline *NPRL3*: NM_001243247.2:c.1216G>A, p.(Pro249Leu) |
| **9** | germline *NPRL3*: NM_001243247.2:c.1216G>A, p.(Pro249Leu) |
| **10** | Somatic NPRL3: NM_001077350.3:c.145_146dup, p.(Ser49Argfs*39) |
| **11** | somatic *MTOR*: NM_004958:c.6644C>A, p.(Ser2215Tyr) |
| **12** | somatic *MTOR*: NM_004958:c.4448G>A, p.(Cys1483Tyr) |
| **13** | somatic *MTOR*: NM_004958:c.7498A>T, p.(Ile2500Phe) |
| **14** | somatic *MTOR*: NM_004958:c.5930C>A, p.(Thr1977Lys) |
| **15** | somatic *MTOR*: NM_004958:c.6644C>T, p.(Ser2215Phe) |
| **16** | somatic *MTOR*: NM_004958:c.7275_7276insCCC, p.(Pro2425_Leu2426insSer |
| **17** | somatic *MTOR*: NM_004958:c.7275_7276insCCC, p.(Pro2425_Leu2426insSer |
